# Striking a balance: the Goldilocks effect of CD8**α** expression on NK cells

**DOI:** 10.1172/JCI182905

**Published:** 2024-08-01

**Authors:** Paarth B. Dodhiawala, Frank Cichocki

**Affiliations:** Department of Medicine, University of Minnesota, Minneapolis, Minnesota, USA.

## Abstract

NK cells are cytotoxic innate immune cells involved in antitumor immunity, and they provide a treatment option for patients with acute myeloid leukemia (AML). In this issue of the *JCI*, Cubitt et al. investigated the role of CD8α, a coreceptor present on approximately 40% of human NK cells. IL-15 stimulation of CD8α^–^ NK cells induced CD8α expression via the RUNX3 transcription factor, driving formation of a unique induced CD8α (iCD8α^+^) population. iCD8α^+^ NK cells displayed higher proliferation, metabolic activity, and antitumor cytotoxic function compared with preexisting CD8α^+^ and CD8α^–^ subsets. Therefore, CD8α expression can be used to define a potential dynamic spectrum of NK cell expansion and function. Because these cells exhibit enhanced tumor control, they may be used to improve in NK cell therapies for patients with AML.

## Regulation of NK cell activation

NK cells are specialized innate lymphoid cells that mediate cellular cytotoxicity without the need for antigen priming (1). Their functional responses are regulated by a balance between signals from activating and inhibitory receptors (2–5). The killer cell immunoglobulin-like receptor (KIR) family encodes both inhibitory and activating members that recognize HLA class I (6). In healthy conditions, inhibitory signals predominate. However, downregulation of HLA molecules on virally infected or malignant cells (7) releases inhibition, tipping the balance toward activation. NKG2A, another inhibitory receptor, binds to HLA-E molecules that are frequently upregulated on the surface of tumor cells to protect them from NK cell–mediated killing (6). NK cells express multiple activating receptors, including natural cytotoxicity receptors and NKG2D, which recognize stress-induced ligands. NK cells also express CD16, which triggers lysis of antibody-coated target cells via antibody-dependent cell–mediated cytotoxicity.

## CD8α expression on NK cells and antitumor immunity

The effect of CD8α on NK cell function is context dependent. CD8α^+^ NK cells exhibit greater cytotoxic function against leukemia cells (8, 9). However, prior work by Fehniger and colleagues using cytokine-induced memory-like NK cells demonstrated that high levels of CD8α on donor memory-like NK cells correlated with treatment failure in patients with relapsed/refractory acute myeloid leukemia (AML) after adoptive transfer (10). In this issue of the *JCI*, Cubitt et al. (11) focused their investigation on conventional healthy NK cells, subtyped as immunomodulatory CD56^bright^ and cytotoxic CD56^dim^, to elucidate the fundamental biological role of CD8α on NK cells. Both NK cell populations expressed CD8α, though levels were higher on CD56^dim^ NK cells. The CD8αα homodimer was the predominant (95%) receptor complex expressed. Single-cell RNA sequencing comparing CD8α^–^ and CD8α^+^ NK cells demonstrated no differences in transcript expression profiles, indicating that CD8α expression does not define a distinct NK cell population in the maturation sequence. To evaluate the effect of CD8α on tumor control, the authors administered CD8α^+^ and CD8α^–^ CD56^dim^ NK cells in a xenogenic K562 leukemia model and observed lower tumor burden in the CD8α^–^ NK cell treatment cohort. These data, supported by additional in vitro experiments, suggest that CD8α dampens the cytotoxic function of NK cells (11).

## CD8α and IL-15 signaling

**Figure 1:**
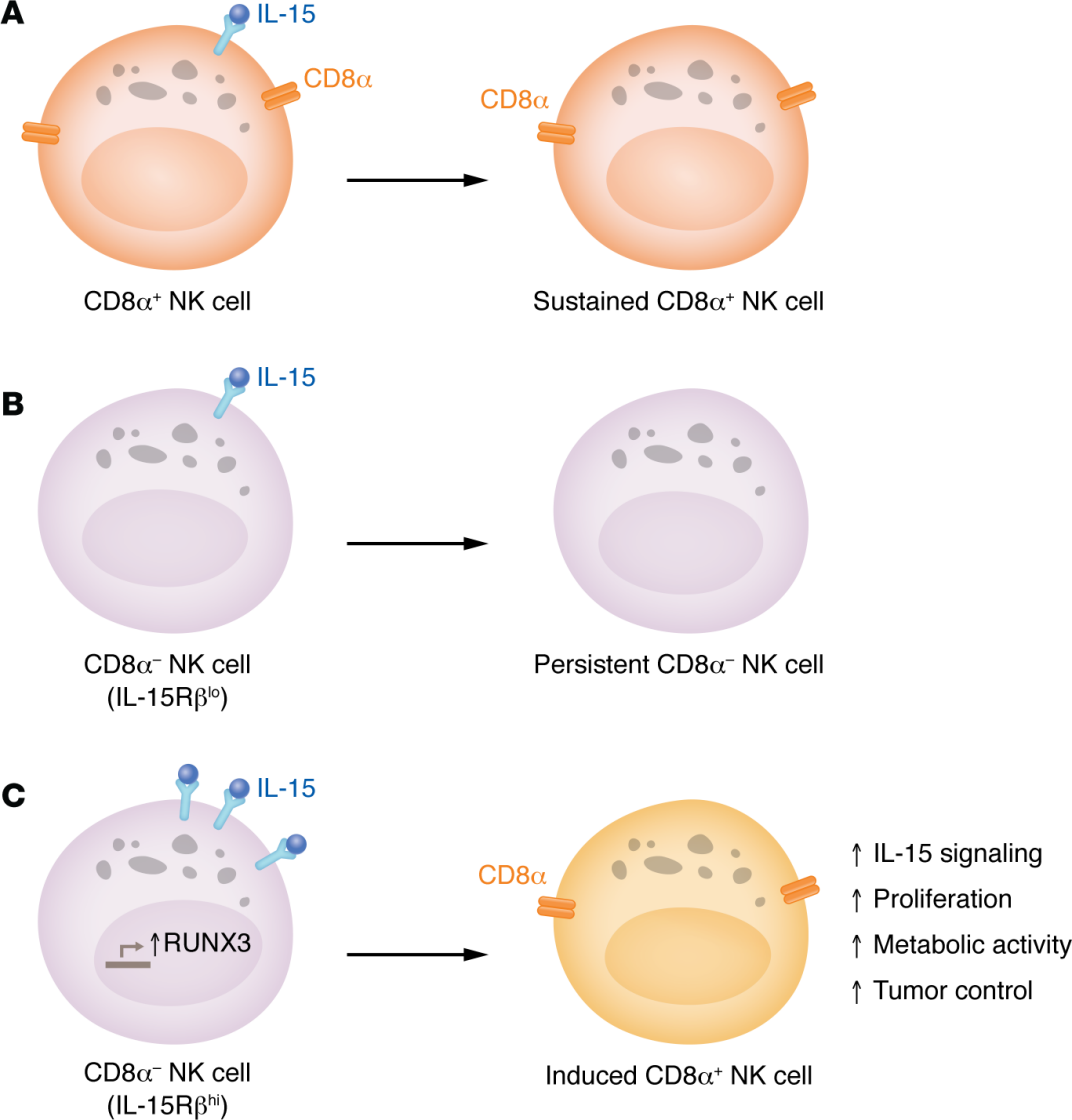
Activation by IL-15 generates induced CD8α^+^ NK cells. Cubitt et al. (11) present three scenarios for human NK cells responding to IL-15. (**A**) In the first scenario, CD8α^+^ NK cells receive an IL-15 signal and become sustained CD8α^+^ NK cells. (**B**) In the second scenario, CD8α^–^ NK cells with low expression of IL-15Rβ are activated by IL-15 and fail to upregulate CD8α, becoming persistent CD8α^–^ NK cells. (**C**) In the third scenario, CD8α^–^ NK cells with high expression of IL-15Rβ upregulate RUNX3 upon IL-15 stimulation and become induced CD8α^+^ NK cells. These cells exhibit several beneficial properties, including enhanced tumor control.

NK cells are dependent on IL-15 for survival and proliferation. IL-15 also primes NK cells and enhances their cytotoxic function against cancer cells (12). Having evaluated the effect of CD8α on cytotoxicity, Cubitt and colleagues then sought to study the potential role of CD8α in IL-15 signaling and proliferation (11). They found that CD8α^–^ NK cells had greater survival and proliferation in response to IL-15 in vitro. In line with these findings, CD8α^–^ NK cells expanded more compared with their CD8α^+^ counterparts in xenogeneic adoptive transfer experiments with IL-15 dosing. The authors also asked whether IL-15 controls CD8α expression and found that IL-15 induced a subset of CD8^–^CD56^dim^ cells to upregulate CD8α, constituting an induced CD8a^+^CD56^dim^ (iCD8α^+^) population (Figure 1). This phenomenon seemed more pronounced in CD56^bright^ cells, though the authors mainly focused on CD56^dim^ NK cells. Interestingly, only CD8αα expression increased, not CD8αβ, suggesting that IL-15 specifically regulates CD8α. Further analysis of this population revealed that iCD8α^+^CD56^dim^ cells exhibited more proliferation in response to IL-15 in vitro and in vivo compared with CD8α^–^ (termed persistent) and original CD8α^+^ (termed sustained) counterparts (Figure 1). Mechanistically, RUNX3, a transcription factor that has predicted binding sites in the *CD8A* locus, showed potential for interaction. CRISPR-mediated deletion of RUNX3 abolished the ability of IL-15 to induce CD8α expression and resulted in decreased expression levels of CD8α in sustained CD8α^+^ NK cells.

To delineate the mechanism upstream of RUNX3, Cubitt and authors investigated components of the IL-15 receptor: IL-15Rα (also known as CD25), IL-2/15Rβ (also known as CD122), and common γ chain (also known as CD132) and activation of downstream effectors. iCD8α^+^CD56^dim^ NK cells had higher expression of CD132 and IL-2/15Rβ and downstream phospho-activated ERK, STAT5, AKT and S6. Furthermore, iCD8α^+^CD56^dim^ NK cells originated from CD8α^–^CD56^dim^ NK cells that had higher preexisting CD122 expression. Induction of CD8α appears to occur downstream of IL-15 signaling, but CD8α itself does not seem to drive proliferation or survival. This premise was supported by the observation that CD8α KO did not augment NK cell expansion or survival with IL-15. Given that IL-15 signaling is known to regulate metabolism in NK cells(11), the authors examined the metabolic profile of iCD8α^+^ NK cells. iCD8α^+^CD56^dim^ NK cells exhibited higher expression of nutrient transport proteins and increased glucose uptake compared with persistent CD8α^–^ and sustained CD8α^+^CD56^dim^ NK cells. Glycolytic capacity correlated positively with CD8α induction, suggesting that greater nutrient uptake and metabolic capacity supports the enhanced IL-15–dependent proliferative capacity of iCD8α^+^ NK cells.

## iCD8α^+^ NK cells in tumor control

Cubitt and authors evaluated the antitumor function of iCD8α^+^ NK cells using a xenogeneic adoptive transfer model with K562 tumor cells (11). After injecting CD8α^+^ and CD8α^–^CD56^dim^ NK cells and monitoring tumor kinetics for 19 days, they isolated 3 NK cell populations: sustained CD8α^+^, persistent CD8α^–^, and iCD8α^+^ NK cells. Ex vivo, iCD8α^+^ NK cells remained most responsive to IL-15 and maintained robust cytolytic activity against K562 when rechallenged. To determine whether CD8α directly affected NK cell activation, the authors examined activating receptor signaling in *CD8A*-KO CD56^dim^ NK cells. *CD8A* KO had minimal effect on lysis of K562 or HL60 cells. Evaluation of receptor signaling showed greater degranulation in *CD8A*-KO cells specifically after NKp30 stimulation. Surprisingly, further investigation did not reveal differences in phospho-activation of signaling molecules downstream of NKp30 engagement. Accordingly, the authors hypothesized that CD8α may augment inhibitory KIR signaling, because KIR and CD8α both bind to HLA molecules. Indeed, calcium flux assays with NKp30 and inhibitory KIR stimulation demonstrated increased NK cell activation in with loss of CD8α. The authors conclude that CD8α suppresses NKp30 NK cell activation by potentiating inhibitory KIR signaling, though it is likely that additional mechanisms are at play.

## Therapeutic implications and future directions

The findings presented in Cubitt et al. provide insights into the dynamic reprogramming of NK cells and raise possibilities for advancing NK cell therapies (11). IL-15 priming increases NK cell cytolytic function (12). However, prolonged IL-15 stimulation results in NK cell exhaustion, characterized by decreased tumor control and diminished mitochondrial metabolic function (13). In this context, it will be useful to determine the trajectory of CD8α expression in relation to NK cell exhaustion. Do NK cells begin as CD8α^–^, acquire CD8α upon IL-15 exposure to become the iCD8α^+^ as described by Cubitt et al., and then ultimately become exhausted, known in this context as “sustained” CD8α^+^? The enhanced metabolic activity of iCD8α^+^ NK cells compared with sustained CD8α^+^ NK cells suggests this possibility, though detailed studies are required. Furthermore, while Cubitt et al. (11) mainly focused on CD56^dim^ NK cells, the magnitude of CD8α induction was greatest in CD8α^–^CD56^bright^ cells. It will be helpful to further characterize iCD8α^+^CD56^bright^ populations and their effect on tumor control, as IL-15–primed CD56^bright^ NK cells can exhibit robust antitumor cytolytic activity (14).

The results of the study by Cubitt et al. (11) have important therapeutic implications. Perhaps CD8α^–^CD56^dim^ or iCD8α^+^CD56^dim^ are favorable for adoptive NK cell therapy in cancer, though additional tumor models need to be tested to prove preclinical efficacy. iCD8α^+^ NK cells displayed a potential memory-like phenotype, as they were able to kill tumor cells when rechallenged. How do iCD8α^+^ NK cells directly compare with CD8α^–^ cytokine-induced memory-like NK cells (15, 16)? Finally, from a mechanistic perspective, further investigation into the role of CD8α is needed to clearly define its activating or inhibitory function on NK cell cytotoxicity. Furthermore, the role of CD8α in antibody-dependent cell–mediated cytotoxicity has not been explored in detail. Yet, for now, the main value of CD8α in NK cells may be the fact that they can define a highly responsive antitumor population that may be exploited to improve NK cell therapies for patients.

## Version 1 08/01/2024

Electronic publication
